# Perceptions of Patients With Chronic Obstructive Pulmonary Disease and Their Physiotherapists Regarding the Use of an eHealth Intervention

**DOI:** 10.2196/humanfactors.7196

**Published:** 2017-09-19

**Authors:** Sigrid Vorrink, Chantal Huisman, Helianthe Kort, Thierry Troosters, Jan-Willem Lammers

**Affiliations:** ^1^ Faculty Chair Demand Driven Care Research Centre for Innovations in Health Care University of Applied Sciences Utrecht Utrecht Netherlands; ^2^ Department of Rehabilitation Sciences KU Leuven Leuven Belgium; ^3^ Division Heart and Lungs Department of Respiratory Medicine University Medical Centre Utrecht Utrecht Netherlands

**Keywords:** telemedicine, self care, physical therapists, pulmonary disease, chronic obstructive

## Abstract

**Background:**

If eHealth interventions are not used (properly), their potential benefits cannot be fulfilled. User perceptions of eHealth are an important determinant of its successful implementation. This study examined how patients with chronic obstructive pulmonary disease (COPD) and their physiotherapists (PHTs) value an eHealth self-management intervention following a period of use.

**Objective:**

The study aimed to evaluate the perceptions of COPD patients and their PHTs as eHealth users.

**Methods:**

In this study, an eHealth self-management intervention (website and mobile phone app) aimed at stimulating physical activity (PA) in COPD patients was evaluated by its users (patients and PHTs). As participants in a randomized controlled trial (RCT), they were asked how they valued the eHealth intervention after 6 months’ use. Interview requests were made to 33 PHTs from 26 participating practices, and a questionnaire was sent to 76 patients. The questionnaire was analyzed in Excel (Microsoft). The interviews with the PHTs and text messages (short message service, SMS) sent between patients and PHTs were transcribed and independently coded in MAXQDA 10 for Windows (VERBI GmbH).

**Results:**

A total of 60 patients with COPD filled out the questionnaire, and 24 PHTs were interviewed. The mobile phone app was used 89.0% (160.2/180 days) (standard deviation [SD] 18.5) of the time by patients; 53% (13/24) of PHTs reported low or no use. Patients scored the ease of use of the app 5.09 (SD 1.14) (on a 7-point scale). They found the presentation of the PA information in the app to be clear, insightful, and stimulating. All PHTs judged the website as explicit and user-friendly but had trouble devising a new PA goal for their patients. Patients mostly sent informative, neutral messages concerning the PA goal, and PHTs sent mostly motivating, positive messages concerning the PA goal. Messages were not perceived as supportive in reaching the PA goal according to the patients. Perceived usefulness of the intervention for the PHTs was the objective measurement of PA, the ability to see PA patterns over time, and the ability to use the intervention as a tool to give their patients insight into their PA. For patients, it was that the intervention supported them in increasing their PA and that it made them feel fitter. Barriers to use of the intervention according to the PHTs were time constraints and financial reasons. Seventy-nine percent (19/24) of the PHTs and 58% (35/60) of the patients mentioned they would be interested in using the intervention in the future.

**Conclusions:**

PHTs and COPD patients had positive feelings regarding the functionality and potential of the eHealth self-management intervention. This paper addresses a number of topics that may aid in the successful development and implementation of these types of eHealth interventions in the future.

## Introduction

eHealth is a relatively new field, and its emergence is causing a shift in health care. Whereas health data have historically been in the hands of health care professionals (HCPs), eHealth apps now provide this information directly to the patient [1]. Furthermore, data collection, insights into the data, and the person that subsequently takes action shifts from the HCP to the patient when using self-management apps.

eHealth has the potential to address the issue of increasing numbers of older adults [2] with relatively fewer HCPs available to provide the required level of service [3]. Moreover, eHealth may also address the increasing number of persons living with chronic conditions such as chronic obstructive pulmonary disease (COPD) [4], who are in need of long-term health care.

In addition to its potential benefits, there are limitations of eHealth that must be mentioned. The limited evidence base is a challenge, as are concerns regarding the privacy of data and the use of eHealth in daily practice. Also, the question of how to engage older adults in eHealth interventions remains an issue [5]. If the interventions are not used (properly), their potential benefits cannot be fulfilled. Furthermore, understanding disease-specific factors to determine how various populations may benefit from eHealth seems important in increasing their use and, subsequently, their efficacy [6]. For example, persons with COPD are generally older adults and are more prone to have a low socioeconomic status [7]. This could negatively impact the (effective) usage of eHealth self-management interventions in this patient group.

User perceptions are an important determinant of the successful use of eHealth. According to the Unified Theory of Acceptance and Use of Technology (UTAUT) model, there are four main constructs that influence the intention to use technology: performance expectancy, effort expectancy, social influence, and facilitating conditions. Additionally, gender, age, voluntary nature of use, and experience with the technology moderate the relationship between the four main constructs and the intention to use [8]. According to the extended expectation-confirmation model in the information technology (IT) domain (extended expectation-confirmation model [ECM]-IT), important predictors of the continued use of technology are perceived usefulness and ease of use, confirmation of expectations, and satisfaction [9]. Continued use of eHealth technologies is especially important when targeting patients with chronic conditions such as COPD. Most eHealth projects begin with little insight regarding user needs and perceptions, which can be an important barrier to implementation [10].

We previously developed an eHealth self-management intervention with the aim to improve or maintain physical activity (PA) in patients with COPD [11]. It comprises a mobile phone app for the COPD patients and a website for their physiotherapists (PHTs). The intervention was tested for efficacy in a randomized controlled trial (RCT) [12] that revealed that the eHealth intervention did not have an effect on PA in this patient group. Based on these unexpected results, the question as to why it was ineffective was raised. This study examined how the users (patients and PHTs) valued the eHealth intervention following a 6-month period of use. The results may help in the future development and successful implementation of similar eHealth self-management interventions.

## Methods

### Study Design

#### Participants

In this study, patients with COPD and their PHTs were asked to evaluate an eHealth self-management intervention. The PHTs worked in primary care physiotherapy practices in the Netherlands and had expertise in treating people with COPD. Patients were diagnosed with COPD, Global Initiative for Chronic Obstructive Lung Disease (GOLD) stage 2 or 3 (forced expiratory volume in 1 s (FEV1) 30 to 80%, FEV1/forced vital capacity (FVC) <70% after bronchodilatation), aged ≥40 years, had completed a pulmonary rehabilitation program of 3 months, and lived independently. PHTs and patients were participants of a RCT [12] and used the intervention for 6 months.

#### eHealth Intervention

The goal of the eHealth self-management intervention is to increase or maintain PA in daily life using step-count goals set by the PHT for each individual COPD patient. The intervention consists of two components: (1) a mobile phone app for patients with COPD for the self-management of PA and (2) a website for PHTs for remote monitoring of their patients.

**Figure 1:**
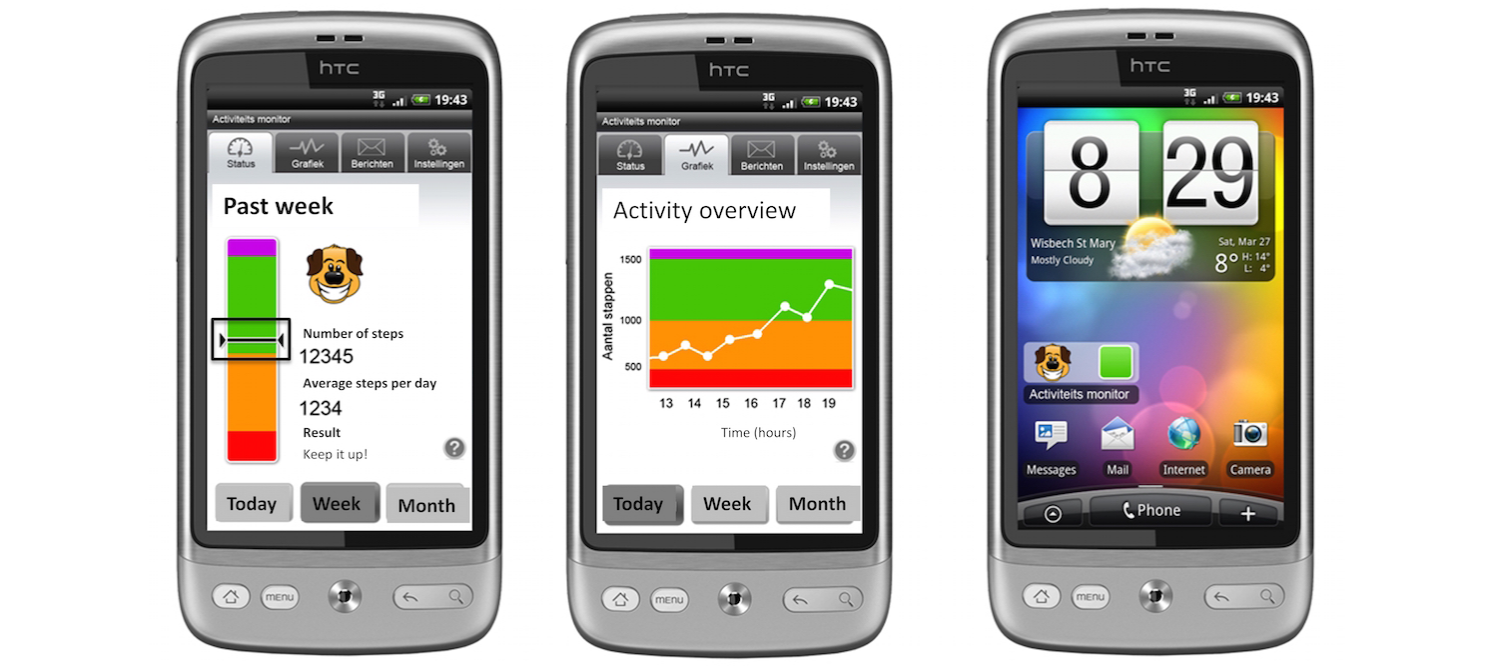
Application. The bar on the left side combines amount and intensity of steps. The physical activity (PA) goal is met when the horizontal stripe (representation of current PA status) is kept in the rising rectangle at all times until the green area is reached. Absolute number of steps and automated encouraging messages linked to current PA progress are also shown.

**Figure 2:**
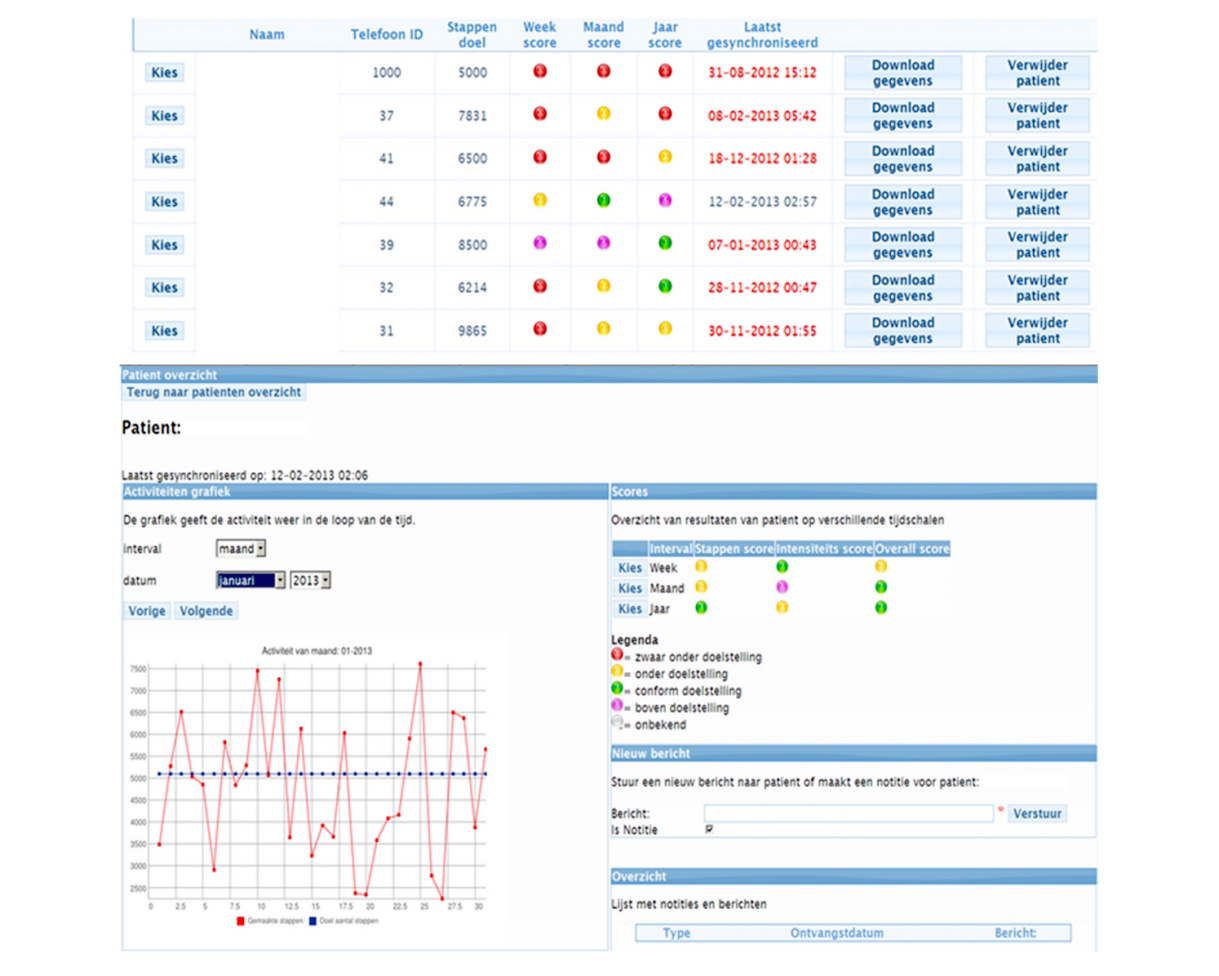
Website for the physiotherapist in Dutch. Above: anonymized overview of the physical activity (PA) goal attainment status of all patients connected to the physiotherapist. Below: detailed PA information of a single subject. The graph on the left shows the PA with the red line and the set PA goal in a blue line. On the upper right scores on PA intensity are shown and on the lower right physiotherapists can sent patients text messages and see an overview of sent and received messages with the current patient.

1. The app (Figure 1) logged and visualized PA in real time in quantitative (steps taken) and qualitative (progress bar) forms as measured by an accelerometer embedded in the mobile phone. Patients were encouraged to reach their personalized PA goal by automatically generated encouraging messages and an emoticon. The automated messages and emoticon in the app were programmed to correspond with the current PA status toward reaching their daily PA goal. The app icon on the home screen indicated current PA status with traffic light colors and an emoticon.

2. PHTs could monitor their patients via the (secure) website (Figure 2) that showed an overview of the PA data from all participants from their practice and a more detailed view of individual patients. The PHT was able to adjust each patient’s PA goal and send group or individual text messages to persuade patients to be physically active and to stimulate them to attain their PA goal [11]. A daily PA goal consisted of the number of steps to be reached, amount of steps per minute that would classify it as an intensive minute of PA, and the number of intensive minutes to be reached. Text messages were synchronized with the mobile phones via an Internet subscription, as were the PA data from the patients to the website of the PHT.

### Methodologies

Participants of the RCT were enrolled in the study for 12 months (from May 2012 to October 2014). This included 6 months of using the intervention and a follow-up measurement at 12 months. After 12 months, interviews were conducted with the PHTs (relating to the total intervention, website, and app), and the patients with COPD received a questionnaire (related to the app). Furthermore, the text messages that were sent during the trial via the website (PHTs) and the mobile phone app (patients) were analyzed.

Interviews were chosen as a method for the PHTs because this group showed low use with the intervention, and it was expected that interviews were the best opportunity to find out the reasons why. The patients were sent a questionnaire to minimize strain on this group, for practical reasons (the group was much larger than the PHTs), and to create a low threshold to participate in this additional study.

**Figure 3:**
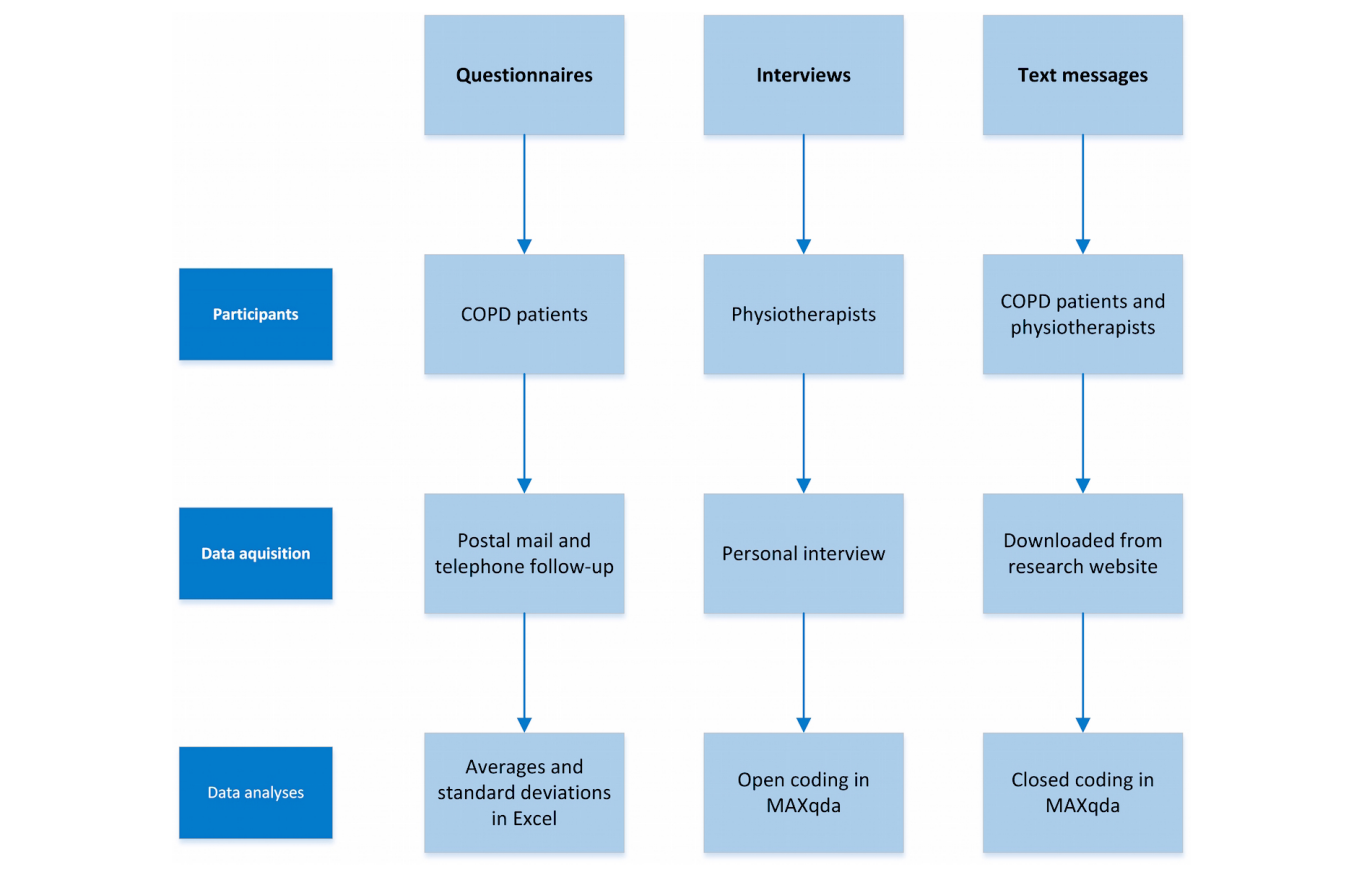
Overview of the methodologies used.

Figure 3 provides an overview of the methodologies used in this study.

#### Physiotherapist (PHT) Interviews

PHTs that treated patients who were included in the intervention group of the RCT were invited for a semistructured interview. The interview structure was based on the rational choice theory [13] and the theory of planned behavior [14]. The first theory states that individuals make choices with the objective of attaining the maximum achievable for themselves or of realizing a certain goal. The second theory accounts for the influence of circumstances and personal and social factors on choices. Interview questions can be found in Multimedia Appendix 1.

The interviews were transcribed and semi open coded by the second author and two research assistants. Based on the interview questions, a basic code list was made. The basic code list consisted of items that were addressed during the interviews such as the app, the website, and the text messages. With the basic code list, the second author and one of the research assistants open coded the interviews until there was a saturation of codes. Open coding was done to ensure that all topics discussed in the semistructured interviews were properly analyzed. The resulting final code list was discussed with the first author. With this final code list each interview was coded twice (by different coders) with the use of MAXQDA 10 for Windows (VERBI GmbH) software package. Differences were discussed between the coders and the first author, after which final decisions were made. The final code list can be found in Multimedia Appendix 2.

#### Patient Questionnaire

After completion of the RCT, questionnaires were sent by postal mail to patients with COPD who participated in the intervention group. A week later, one of the researchers discussed all of the questions with the patients during a phone appointment to ensure that they were properly understood. The questionnaire was composed of three existing questionnaires: the Usefulness, Satisfaction, and Ease of Use (USE) questionnaire on usability [15], which results in total scores for the domains of usability, ease of use, ease of learning, and contentment; the Florida State University (FSU) mobile device feedback preferences scale; and the FSU physiological monitoring privacy scale (inspired by Beach et al [16] and Kwazney et al [17]). Eight out of 38 questions from the USE questionnaire, 15 from the FSU feedback scale, and 14 from the FSU privacy scale were slightly adjusted to be specifically directed toward the intervention at hand. We added 6 questions regarding circumstances influencing the ability to reach the PA goal and whether patients would like to continue to use the intervention (questions 68-73). The questionnaire can be found in Multimedia Appendix 3. The results of the USE questionnaire were summarized per its instructions [15]. For the other results, averages and standard deviations (SDs) were computed in Excel (Microsoft). For the 8-point scales (0-7), a score of 3.5 or higher was seen as satisfactory, and for the 7-point scales (1-7), a score of 4 or higher was seen as satisfactory.

#### Text Messages and Use of the Intervention

The frequency of text messages sent between the PHTs and the COPD patients was recorded. Content was analyzed by coding similar to the interviews. However, three final code lists were established in advance by the second author after a read through of the messages and discussion with the third author; one for tone (positive, negative, and neutral), type (motivating, informative, fun or social, and question), and topic (PA goal, mobile phone or app, health, study related, and other). Tone was chosen to give insight into the ways in which patients were motivated by their PHTs to reach their PA goal. The second author and a colleague independently coded the messages, after which differences were discussed with the third author, and final decisions were made.

Adherence to the intervention was measured as the percentage of days that the intervention was used and as the percentage of days that the PA goal was attained.

### Ethics

According to the Central Committee on Research Involving Human Subjects, interviews or questionnaires do not require ethics approval unless the questions are very *detailed*, *burdensome*, or *intimate* [18].

**Table 1:** Demographics of the chronic obstructive pulmonary disease (COPD) patients (N=60).

Outcome		Mean (SD) or number
Age in years, mean (SD)		62 (8)
**Gender**	Female	25
	Male	35
Body mass index (kg/m2), mean (SD)		27 (5)
Forced expiratory volume in 1 second (liters), mean (SD)		1.71 (0.60) (59 [SD 20]% predicted)
Forced vital capacity (liters), mean (SD)		3.61 (0.95) (99 [SD 19]% predicted)
6-minute walking distance (meters), mean (SD)		486 (84) (83 [SD 15]% predicted)
Average steps/day (weekday), mean (SD)		5980 (3035)

## Results

### Demographics

**Figure 4:**
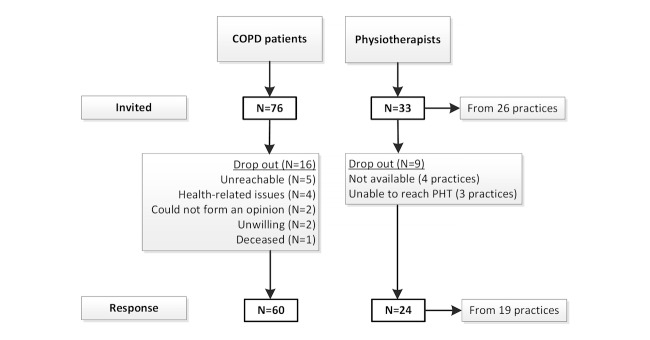
Consort flowsheet participants.

PHTs from 26 physiotherapy practices (with patients in the intervention group during the RCT) were invited for the interviews. A total of 76 COPD patients (that were randomized into the intervention group) were invited to fill out the questionnaire (Figure 4). The questionnaire for the patients did not contain missing values.

The average age of the participating PHTs was 44 years (SD 11). In total, 16 females and 8 males were interviewed. Demographics of the participating COPD patients and their baseline measurements during the RCT [12] are shown in Table 1.

The results are presented in five segments: the use of the intervention, the app, the website, text messages, and the results of the eHealth intervention in general. Multimedia Appendix 4 provides more detailed results of the questionnaire for the patients with COPD regarding use, privacy, feedback preferences, and personal circumstances. Below, the most important findings are described. At the end of the results, the key findings are summarized in Table 6.

### Use

Patients with COPD used the eHealth self-management app on 89.0% (160.2/180) (SD 18.5) of the days that it was in their possession (6-month period). They attained their personal PA goals on 33.8% (61/180) (SD 16) of these days [19]. The reported use of the website by PHTs varied from 5-60 min per session. Nine practices used it every week, 3 used it every other week, 4 used it mostly at the start of the RCT, and 3 did not use it at all. Ten practices mentioned having spent barely any time on the website. Three PHTs scheduled time in their agenda to use the website. PHTs mentioned that patients’ and their own motivation to use the intervention diminished over time.

### Application

Patients considered the app to be fairly easy to learn and use (Table 2). Training on the use of the app was not reported as highly necessary. The presentation of the PA information in the app was considered to be clear, insightful, and stimulating. Desired options included the possibility to measure cycling, swimming, and distance walked.

Patients liked the fact that PA was presented in steps, thought the bar and graph provided extra insight into their PA status, and that the emoticon and written advice were stimulating. The widget on the home screen clearly stated current PA status, and they thought it was pleasant to have various choices of emoticons. Patients think their PA information should be visible to them and not only to their PHT.

Health status, energy level, personal circumstances, and timeconstraints did not negatively influence patient ability to reach the PA goal (Multimedia Appendix 4).

**Table 2:** Application: Usefulness, Satisfaction, and Ease of use (USE) questionnaire scores (mean [SD]). Scores range from 0-7 (0: totally disagree and 7: totally agree).

Outcome	Mean (SD)
Ease of learning	5.55 (1.46)
Ease of use	5.09 (1.14)
Contentment	5.06 (1.54)
Usability	4.97 (1.32)

**Table 3:** Feedback on website by physiotherapists.

Remark	Number of practices
There should be a mobile version of the website to use on your mobile phone	3
Integrate into standard patient software	3
Patients should receive message notifications	2
Show intensity scores also in patient overview	2
Remove year scores (not relevant)	2
Meaning of scores not always clear (colors)	2
Add Borg score (rating of perceived exertion)	1
Graphs were difficult to read	1
Show week scores in patient overview	1
Show medication use and other types of exercise on the website	1
“I don’t trust the intensity scores”	1

PHTs mentioned that the app was explicit and user-friendly for their patients. Six PHTs mentioned that some of their patients had trouble sending text messages as a result of the small keyboard or overlooked the possibility. Nine PHTs mentioned that there were differences among the patients with regards to digital skill level. Personal instruction on the use of the intervention was deemed important, especially for older users. PA status was often viewed by patients and was regarded as stimulating. PHTs suggested that patients should have the option to indicate if they were having a bad day and, subsequently, that their daily PA goal would be adjusted accordingly.

### Mobile Phone

Patients scored the ease of use of the mobile phone as 5.7 (SD 1.65) (on a 7-point scale). A total of 32% (19/60) of patients owned a mobile phone before the start of the study, and 18% (11/60) purchased one after the study. Technical failure of the mobile phone or app or forgetting to bring the phone was not a major issue (Multimedia Appendix 1).

Six PHTs mentioned that the use of the mobile phone, as well as continuously wearing the mobile phone, was considered troublesome for one of their patients (but not for the rest of their patient group). Personal instruction regarding the use of the mobile phone was considered important, but they found it important not to give too much information at one time.

### Website

All interviewed PHTs considered the website to be explicit and user-friendly. They used it to view PA data, adjust PA goals, and send text messages. However, reported use was low due to time constraints. The additional log-in was considered tedious, and the PHTs mentioned that a website that could be incorporated into their usual patient software would be better.

Five practices adjusted PA goals via the website, and one adjusted PA goals via the researchers, whereas seven reported that they did not adjust PA goals. Devising a new goal was considered difficult, especially regarding PA intensity, which was seen as an important outcome and thought to predict exacerbations. However, the intensity scores of the daily PA goal (see Methods) were not always well understood.

PHTs noted that they would like to receive a notification when a patient was deteriorating over a longer time period. Table 3 shows PHTs feedback on specific website items and suggestions for improvement.

### Text Messages

Fifteen practices sent text messages to their patients. Group messages were sent by 8 practices. Thirty-five patients sent text messages to their therapists. Information on the number of messages sent between the PHTs and the patients can be found in Table 4.

**Table 4:** Number of messages sent by physiotherapists and patients.

Users	Text messages sent	Erroneous messages
	Type/N	N (% of total)
Physiotherapists	Personal: 382 Group: 12	41 (10)
COPD^a^ patients	162	16 (9)

^a^COPD: chronic obstructive pulmonary disease.

**Table 5:** Types of messages sent. Results are given as frequencies and percentages of total messages sent.

Type of message	By the physiotherapists (N)	Percentage of total messages (%)	By the COPD^a^ patients (N)	Percentage of total messages (%)
Motivating	241	56	0	0
Informative	68	17	117	66
Question	43	10	11	6
Fun or social	20	8	34	19
Total	372	100	162	100

^a^COPD: chronic obstructive pulmonary disease.

The messages sent by the PHTs mostly concerned the PA goal (72.8%, 287/394). The remaining messages were related to the mobile phone or app (10.9%, 43/394), the study (7.1%, 28/394), health (4.5%, 18/394), or other topics (3.8%, 15/394). For the patients, this was more evenly divided, with 30.2% (49/162) of the messages concerning the PA goal; 19.7% (32/162), the mobile phone or app; 9.3% (15/162), the study; 19.1% (31/162), health; and 22.2% (36/162), other topics. PHTs mostly sent motivating messages, whereas patients mostly sent informative messages. Table 5 presents the distribution of the types of messages sent. The tone of the messages sent by the PHTs was mostly positive (63.9%, 252/394), followed by neutral messages (36.0%, 142/394), and 2 negative messages (0.5%). For patients, positive (55.0%, 88/160) and neutral (43.1%, 69/160) messages were more evenly divided. They sent 15 (3%) negative messages.

Patients mentioned that sending messages to and receiving them from the PHT was rare and was not seen as supportive in reaching their PA goal (Multimedia Appendix 4). PHTs from 6 practices explained that they used text messages to inform patients, to motivate them, and to determine the reason why the PA goal was not met. One PHT emailed patients instead of texting. During the RCT, the PHTs contacted all subjects but mentioned that if the intervention was implemented, they would contact only those who did not reach their PA goals.

### eHealth Self-Management Intervention in General: Perceived Usefulness, Applicability, and Privacy

#### Perceived Usefulness

PHTs mentioned that the intervention provided them insight into the objective PA data of their patients outside the clinical setting, whereas previously they had to rely on the account of the patient. This was regarded by them as a major advantage. It also enabled them to see patterns in PA. Nine PHTs mentioned that the ups and downs in the PA of patients with COPD are important to monitor in light of exacerbations. The data can be used to start a conversation with the patient about their PA level and to give them insights and tips. One PHT mentioned that his patients learned how far they needed to walk to reach their PA goal during the intervention period and continued to do so after the study ended. Nine PHTs found it pleasant and necessary to follow patients after pulmonary rehabilitation (PR), whereas two PHTs did not see this as a task for the PHT. Patients thought that the eHealth intervention helped them to increase their PA and made them feel fitter. It was rewarding for patients to reach their PA goal (Multimedia Appendix 4).

#### Applicability

PHTs from 15 practices mentioned they would be interested in using the intervention, provided that it proved effective and that the helpdesk would remain available. Two practices stated that they would not be interested in using the intervention. Two practices were unclear on this matter. Additionally, 58% (35/60) of the patients mentioned that they would like to start using the intervention again. The PHTs believe that the eHealth intervention may be useful in preventing relapses and subsequent repeated PR. PHTs from 8 practices thought that the intervention should already be used during PR, and 4 practices preferred to start after the program.

PHTs believe that face-to-face contact every 2-3 months is necessary, in addition to monitoring from a distance. Additionally, the use of the intervention should be individually tailored to each patient.

There were questions regarding the financing of the intervention. PHTs were concerned that they would not be paid by health care insurers because monitoring is not seen as a consultation; therefore, expenses cannot be claimed. Additionally, they considered it an issue that not all patients owned a mobile phone.

#### Privacy

Patients reported that they did not worry about privacy with regards to their PA data. Interested parties such as family and PHTs are welcome to access the data; however, local authorities are not. It is important that patients have control over who can see their data (Multimedia Appendix 4). Two PHTs mentioned that privacy is an important consideration when using eHealth.

**Table 6:** Key findings

Topic		Patients	Physiotherapists
		The intervention was used on 89.0% (160.2/180) (SD^a^18.5) of the days in their possession	10 out of 19 practices spent little time on the intervention
**App**			
		Easy to learn and use Explicit and user-friendly to patients	
		Training not necessary	Training necessary
		Clear, insightful, and stimulating	
**Mobile phone**				
		Easy to use	Use of and continuously wearing the mobile phone troublesome for a few patients
		32% (19/60) owned a mobile phone, 18% (11/60) purchased one after the RCT^b^	
**Website**			
			Explicit and user-friendly
			Used to look at PA^c^data, adjust PA goals, and to send messages
			Setting PA goals was considered difficult
			Reported low use was attributed to time-constraints
			Tedious additional log-in
**Text messages**			
		Sent mostly informative, neutral messages concerning the PA goal	Sent mostly motivating, positive messages concerning the PA goal
		Messages were not perceived as supportive in reaching the PA goal	
**eHealth self-management intervention general**			
	Perceived usefulness	Felt it helped to increase PA	Measure of objective PA data outside the clinical setting
		Made them feel fitter	Ability to see patterns in PA (to monitor exacerbations)
			Tool to start a conversation about PA with the patient
	Applicability	58% (35/60) would like to continue to use the intervention	15 out of 19 practices were interested to use the intervention
			Could be useful in preventing relapse
			Financing concerns
			Face-to-face is necessary in addition to monitoring
			Intervention should be individually tailored to the patient
	Privacy	Important to have control over the distribution of their data	Important aspect to keep in mind when working with eHealth

^a^SD: standard deviation.

^b^RCT: randomized controlled trial.

^c^PA: physical activity.

## Discussion

This study evaluated the perceptions of patients with COPD and their PHTs and the text messages both groups sent regarding the use of an eHealth self-management intervention aimed at stimulating PA in patients with COPD.

### Principal Findings

#### Use

Measured use among patients was high, whereas PHTs reported low use. Barriers to use the intervention according to the PHTs were time constraints and financial reasons. Implementation of the intervention in daily practice was challenging. PHTs suggested various features that may enable its use such as a mobile phone app for the PHT, a notification when a patient deteriorates, and a website that is incorporated into the standard patient software.

#### Application

Patients scored the mobile phone and app satisfactory with respect to ease of learning and use. Contentment with and usability of the app was also scored as satisfactory.

Patients were disappointed that the app could not measure cycling or swimming and that it did not capture the intensity of walking the stairs. There were quite a few patients who cycle a lot and were disappointed when this was not added to the overall PA goal attainment. For COPD patients living in countries with a strong cycling tradition, this activity is seen as an important part of PA, whereas it is not relevant for individuals living in other countries [20]. This shows that nationality or culture can also influence the needs and wishes of the end user and should be considered.

As of the time of the study, battery capacity was too low to add global positioning system (GPS) measurements or other features that could measure these activities. As the development rate of mobile phone technologies and accompanying batteries is high, this seems likely to be possible in the near future. For example, identifying the activity of “walking the stairs” has recently become possible [21]. Also, waterproof mobile phones are now available, so swimming can be measured as well [22].

According to the PHTs, there were some patients who had trouble using the mobile phone. With proper instruction and training, mobile phone and other technology use in older adults has not shown to pose many problems [23].

Face-to-face instructions are usually preferred by older adults [24]. The PHTs warned us of an information overload at the initial instruction for the patients. We may have provided too much information at once. The written instructions and help desk were helpful in this regard.

One-third of the patients owned a mobile phone, and 18% (11/60) purchased one after the study. This was in 2012, 2013, and 2014 when mobile phone use among older adults (65+ years) in the Netherlands was 11, 17, and 26%, respectively [25]. This result, combined with the high use rate in patients, is promising in light of mobile phone–based eHealth self-management interventions for older adults and patients with COPD in particular.

#### Website

PHTs considered the website to be explicit and user-friendly. Several suggestions were made to improve usability of the website (Table 3). Important with regard to the aim of the intervention were the results on setting or adjusting the PA goals.

Only 6 out of 19 practices that were interviewed adjusted the PA goals of their patients. If there were patients in the other practices that had trouble achieving their PA goals or their goals were too easy for them, they may have been demotivated. Three PHTs mentioned that they found it difficult to set a new goal. Despite the personal instructions for the PHTs regarding the intervention, some did not completely understand the intensity scores of the PA goal. Furthermore, as there are no COPD-specific PA guidelines available, PHTs had to rely on their own practice-based expertise. This can be difficult, especially because minor changes in the frequency, intensity, and time of general PA guidelines for older adults can have major consequences for patients with COPD regarding their ability to comply with these guidelines [26]. Furthermore, automated PA goal setting with the option of an override by the PHT could prove the best option.

The PHTs mentioned that it would be beneficial if patients had the opportunity to indicate whether they are having a bad day. During the measurements, a few patients also mentioned that there were days that they wanted to attain the PA goal but were too tired or were too affected by dyspnea to do so. The option to adjust daily PA goals to account for fluctuating physical capacity may improve goal attainment in this patient group. As a result, positive feedback may increase, and patients may be more motivated to use the intervention long-term.

#### Text Messages

The text messaging function was not used to its full potential. Only 15 out of 26 practices sent messages. Additionally, only 10 group messages were sent. Similar to the PHTs, there was a large portion of patients who did not use this function (53%, 32/60). Patients did not perceive messaging as supportive in reaching their PA goal, which is not surprising considering its low use rate. Low use could stem from a suboptimal interface (eg, the letters on the mobile phone keyboard were small), as we found that both PHTs and patients sent erroneous messages in approximately 10% (39/394) of cases. One PHT used email instead of messaging, choosing the technology she is more familiar with.

In looking at the correct messages, we see that the messages sent by the PHTs mainly focused on the PA goal and were positive and motivating. This was the intention of this aspect of the intervention. Perhaps if all PHTs had sent these messages, patient PA outcomes would have improved. Automatic reminders could assist in this regard. Responses from patients would likely have been higher as well. On the other hand, 80% (48/60) of patients were still seeing their PHT once or twice a week. During these meetings, the PA measurements of the intervention were discussed and patients motivated. This would have rendered (some) messages superfluous.

#### Intervention General

Interestingly, patients thought that the intervention helped them to increase their PA and made them feel fitter. However, the data from the RCT does not show a difference in PA over time compared with the usual care group, and PA actually diminished in both groups equally over the 1-year study duration [12].

Although, in general, the reported use of the website and messaging function was low, PHTs were positive about the functionalities of the intervention. Thus, one could argue it was not the intervention itself but rather its cumbersome implementation that caused the low use by PHTs. Financing concerns were expressed regarding implementation. These may stem largely from a lack of awareness regarding the financing options of the Dutch Health Authority concerning eHealth. Educating PHTs on funding for eHealth could remove this barrier for use.

PHTs mentioned that face-to-face contact every 2-3 months is necessary, in addition to long-term monitoring. eHealth should be seen as an addition to current health care instead of as a replacement. Clearly indicating this to HCPs might help in eHealth acceptance since it can be seen as a threat to their job.

In the introduction, we mentioned that perceived usefulness and ease of use, confirmation of expectations, and satisfaction with the technology are important predictors of continued use [9]. Perceived usefulness for the PHTs was that the PA data was objectively measured, the ability to see PA patterns over time, and that they could use this data to give their patients insight in their PA. They mentioned that they used the intervention as a tool to start a conversation with their patients about their PA. For patients, it was that the eHealth intervention helped them to increase their PA and made them feel fitter. This shows that the intervention has the potential to help patients self-manage their PA. Reported ease of use by patients and PHTs was satisfactory with regard to the app and the website. We cannot draw any conclusions regarding confirmation of expectations because this was not measured at the start of the RCT. For a measure of satisfaction in patients, we can examine the contentment score of the app, which was adequate. For PHTs, this is more difficult because they reported low use. However, they were positive on the functionality and potential of the eHealth intervention.

### Limitations

We thought it would be important to pay extra attention to the patients to ensure that they would use the intervention. The PHTs were the ones who initially signed up their practice to participate in the RCT and were thus thought to need less attention. They were given one face-to-face instruction session, written instructions, and access to a helpdesk. In hindsight, they may have needed more prompting and training to use the intervention. For successful use of eHealth interventions, HCPs need new competencies such as composite skills and technology-specific competencies [27]. Inadequate training and education of HCPs can function as a barrier to implementation [10]. Coaching skills, the ability to combine clinical experience with technology, communication skills, clinical knowledge, ethical awareness, and a supportive attitude are seen as core competencies needed by HCPs to effectively use eHealth technologies [28]. Future studies may benefit from training the HCPs to improve these competencies.

PHTs were interviewed by a member of the research group. This may have led to more favorable answers toward the intervention to please the researchers. The same holds true for the patients who were telephoned to ensure that they understood all of the questions in the questionnaire. Furthermore, the PHTs (9) and patients (16) that were not interviewed or did not fill out the questionnaire may have had a lower use rate and more negative opinions.

The interviews and text messages were coded by the research team and one colleague. To avoid any bias, it would have been preferable if coding was done by people without further knowledge of the study.

Because the RCT had a follow-up measurement at 12 months, interviews and questionnaires were conducted 6 months after the period of use. This could have caused recall bias.

### Comparison With Prior Work

For eHealth self-management apps, we found user evaluation studies for diabetes [29-31] and dementia [32]. Bender et al [33] performed a systematic review on mobile phone apps for the prevention, detection, and management of cancer. They concluded that even though there are hundreds of cancer-focused apps, there is a lack of evidence on their utility, effectiveness, and safety. This seems to hold true for COPD-focused apps as well. However, we did find some user evaluations of Web-based applications for COPD (in-home PR [34,35] and a self-management support application [36]). One study evaluated the use of a similar mobile phone-based app to stimulate PA in COPD [37]. Eighty-eight percent (53/60) of the patients used it until the end of the intervention period, in spite of high numbers of technical problems. Our study similarly showed high adherence rates to the intervention. Another similarity was that the monitoring HCPs struggled to fit the extra consultations into their busy daily practice.

As in this study, other studies also stress the importance of training of patients [29] and HCPs [38] on the proper use of the eHealth technology. Besides training, studies propose several important elements to incorporate in the design of eHealth apps, such as automatic data transfer when possible, motivational and visual user interfaces, peer support, individual tailoring, and considerable health benefits in relation to the effort required [30,35]. Furthermore, Bitterman et al [39] mention that it has to be taken into account that, compared to the use of medical equipment in the standardized hospital environment used by experienced and well-trained HCPs, users of home medical devices and services are a heterogeneous, primarily nonprofessional group that operate the device in an unpredictable and uncontrolled environment.

Often, some patients benefit more from eHealth self-management apps than others [29]. Jalil et al [29] propose the Clinical User-Experience Evaluation (CUE) methodology to unpack the variations in outcome of individual patients using the technology. This is a three-step process where first, the user uses the device while using the “think-aloud” method; second, the user is interviewed; and third, is given an anonymous survey to express opinions without reservations. Having a standardized method might assist researchers in performing more (comparable) user evaluations.

### Conclusions

PHTs and patients were positive regarding the functionality and potential of the eHealth self-management intervention. Patients used the intervention on 89.0% (160.2/180) of the days that it was in their possession. Fifty-three percent of PHTs reported low or no use. Patients rated the mobile phone and app as easy to use. They found the presentation of the PA information in the app to be clear, insightful, and stimulating. PHTs considered the website to be explicit and user-friendly. Perceived usefulness of the intervention for the PHTs was the objective measurement of PA, the ability to see PA patterns over time, and the ability to use the intervention as a tool to give their patients insights into their PA. The patients reported that it supported them in increasing their PA and made them feel fitter.

Fifty-eight percent (14/24) of PHTs and 47% (28/60) of patients used the messaging function. PHTs sent mostly motivating, positive messages concerning the PA goal, whereas patients sent mostly informative, neutral messages concerning the PA goal. The messages were not perceived as supportive in reaching the PA goal by patients.

Barriers to use of the intervention according to the PHTs were time constraints and financial reasons. Devising a new goal was considered difficult. However, 79% (19/24) of the PHTs and 58% (35/60) of the patients mentioned they would be interested in using the intervention in the future.

## Appendix

Multimedia Appendix 1 Interview questions physiotherapists.

Multimedia Appendix 2 Final code list.

Multimedia Appendix 3 Patient questionnaire.

Multimedia Appendix 4 Results patient questionnaire.

## Abbreviations

COPD: chronic obstructive pulmonary disease
ECM-IT: expectation-confirmation model in IT domain
FSU: Florida State University
GPS: global positioning system
HCP: health care professional
IT: information technology
JMIR: Journal of Medical Internet Research
PA: physical activity
PR: pulmonary rehabilitation
PHT: physiotherapist
RCT: randomized controlled trial
SD: standard deviation
USE: Usefulness, Satisfaction, and Ease of Use questionnaire
UTAUT: Unified Theory of Acceptance and Use of Technology
