# Supplementary material for: Perceptions of Patients With Chronic Obstructive Pulmonary Disease and Their Physiotherapists Regarding the Use of an eHealth Intervention
Source: JMIR Hum Factors. 2017 Sep 19;4(3):e20. doi: 10.2196/humanfactors.7196 (PMC5627045; doi:10.2196/humanfactors.7196)
Supplement: Multimedia Appendix 1 [file humanfactors_v4i3e20_app1.pdf]

Multimedia Appendices Vorrink et al.

Multimedia Appendix 1

Interview questions physiotherapists

Interview Structure – Open interview

Requirements: voice recorder (borrow from IT helpdesk) and smartphone with application.

Phase of consideration

Starting question:

“You have participated in the eHealth study to increase/retain physical activity of COPD patients after a rehabilitation period, could you tell us how you came to this decision?”

The answer must contain:

Rational factors

1) What did you do to try and increase/retain physical activity of COPD patients after a period of pulmonary rehabilitation in the past?
2) Beforehand, what were the perceived advantages and disadvantages of the eHealth intervention and which were the most important in deciding to participate in the study?
3) Which aspects did you miss in the application and/or website?

Personal factors

1) How were you informed about the study and was this information sufficient to use the intervention?
2) How much time were you given to use the website?
3) Were there aspects you missed in the given information?

Social factors

1) Who worked with the website within the physiotherapy practice?
2) What did these persons think about the website?
3) Do you feel communication between the researchers and these persons was sufficient?

Phase of use

Starting question:

“You have coached/monitored patients with the eHealth intervention after a rehabilitation period, could you indicate how you experienced this coaching/monitoring?”

The answer must contain:

Rational factors

1) Did you have the impression that you can positively affect physical activity of COPD patients by using the eHealth intervention?
2) Which aspects did you miss when coaching/monitoring your patients with the eHealth intervention and how did you solve this?

Personal factors

1) Did you have enough time, knowledge and skills to monitor the patients and intervene with the eHealth intervention?
2) What problems were you facing when you used the website?

Social factors

1) Who have been involved with the coaching/monitoring of the patients?
2) How did these persons rate the eHealth intervention?
3) What reactions did you hear about the intervention from your patients?

Final evaluation

Start question:

"Would you like to use the eHealth intervention again in the future and why? How can we improve the eHealth intervention?"

- ➔ Look at the pictures and ask for every aspect whether this is good or what can be done better. Also use the smartphone to show the application.

Rational factors:

1) Could you coach/monitor your patients enough by using the eHealt intervention?
2) What have you missed in coaching/monitoring your clients?

Applicatie

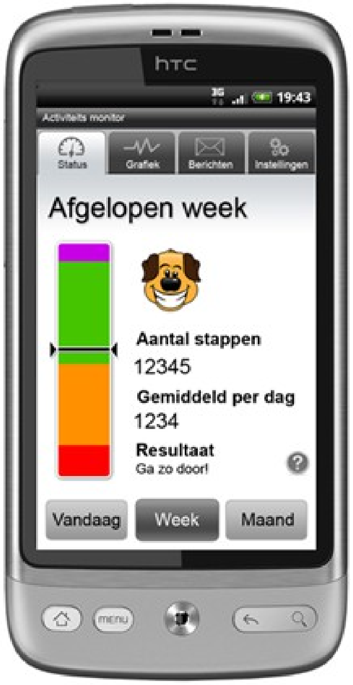

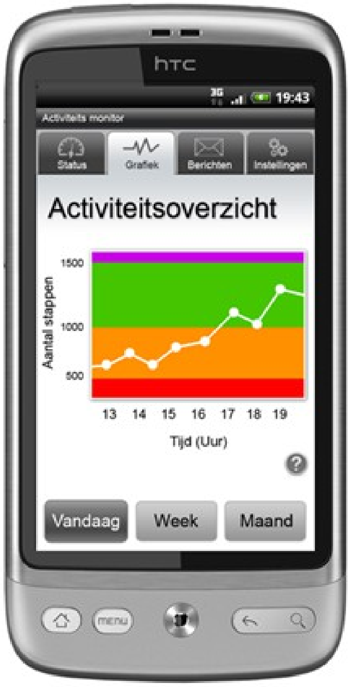

Website

	Naam	Telefoon ID	Stappen doel	Week score	Maand score	Jaar score	Laatst gesynchroniseerd		
Kies	Test simulator	1000	5000				31-08-2012 15:12	Download gegevens	Verwijder patient
Kies		37	7831				08-02-2013 05:42	Download gegevens	Verwijder patient
Kies		41	6500				18-12-2012 01:28	Download gegevens	Verwijder patient
Kies		44	6775				12-02-2013 02:57	Download gegevens	Verwijder patient
Kies		39	8500				07-01-2013 00:43	Download gegevens	Verwijder patient
Kies		32	6214				28-11-2012 00:47	Download gegevens	Verwijder patient
Kies		31	9865				30-11-2012 01:55	Download gegevens	Verwijder patient
Kies		2453230033	1282				12-02-2013 06:29	Download gegevens	Verwijder patient
Kies		36	2541				12-02-2013 05:49	Download gegevens	Verwijder patient
Kies			9999				30-01-2013 03:39	Download gegevens	Verwijder patient
Kies		42	9558				11-02-2013 17:08	Download gegevens	Verwijder patient
Kies		34	5000				18-06-2012 08:42	Download gegevens	Verwijder patient
Kies		40	10700				07-01-2013 05:27	Download gegevens	Verwijder patient
Kies		43	8500				07-01-2013 01:35	Download gegevens	Verwijder patient
