# Supplementary material for: Perceptions of Patients With Chronic Obstructive Pulmonary Disease and Their Physiotherapists Regarding the Use of an eHealth Intervention
Source: JMIR Hum Factors. 2017 Sep 19;4(3):e20. doi: 10.2196/humanfactors.7196 (PMC5627045; doi:10.2196/humanfactors.7196)
Supplement: Multimedia Appendix 2 [file humanfactors_v4i3e20_app2.pdf]

Multimedia Appendix 2

Final code list

1. Application (app)
    a. General
    b. Tab status
        i. PA goal
    c. Tab graph
    d. Tab messages
    e. Tab settings
    f. Experience patient
    g. Areas of improvement
2. Website/portal
    a. General
    b. Overview page
        i. Messaging
        ii. Columns on the page
        iii. "Traffic light colors"
    c. Patients overview
        i. Graph
        ii. Scores
        iii. Messaging
        iv. Set and adjust PA goal
        v. Intensity score
    d. Areas of improvement
    e. Use by physiotherapist
3. Experiences smartphone
    a. Experiences patient
        i. Usability
        ii. Easy anxiety
    b. Experiences physiotherapist
4. Reasons of participation
    a. Approached or own initiative
    b. Motive of participation
        i. Expect benefits
        ii. Expect disadvantage
5. Provision of information from the researchers
    a. Before the start of the research
        i. Inclusion of patients
            1. Inclusion and exclusion criteria
        ii. Website/portal
        iii. Application
        iv. Research progress
    b. During the research
        i. Website
        ii. Application
        iii. Research progress
            1. Measurement moments
            2. Problems
            3. Support of researchers

6. Time allocation by physiotherapist
    a. How much time is spent
    b. Scheduled vs. not scheduled
    c. Individually or with colleagues
7. Applicability intervention in the future
    a. Expense to use de intervention
    b. Motive for using the intervention in the future
8. Practice information
    a. Personal information physiotherapist
    b. Team
    c. Usual care COPD patients
9. General information COPD
10. Research
    a. Information about patients
    b. Results measurement moments
    c. Number of patients
    d. Support of patients with intervention
