# Supplementary material for: Perceptions of Patients With Chronic Obstructive Pulmonary Disease and Their Physiotherapists Regarding the Use of an eHealth Intervention
Source: JMIR Hum Factors. 2017 Sep 19;4(3):e20. doi: 10.2196/humanfactors.7196 (PMC5627045; doi:10.2196/humanfactors.7196)
Supplement: Multimedia Appendix 3 [file humanfactors_v4i3e20_app3.pdf]

Multimedia Appendix 3

Patient questionnaire

Dear sir/madam,

You have participated in the study titled: “electronics in health care to improve COPD care”. During the study you used a smartphone (mobile phone) with an application (the program that counted your steps). We would like to know what your experiences were with the smartphone and the application. To do this we have created a questionnaire. We would like to ask you to fill this out. In total there are 73 questions and to answer them will take 20 minutes of your time. Some questions have additional space to write down a remark. Remarks can also be placed at the end of the questionnaire. With the outcomes of this questionnaire we can improve the application. When a question is unclear you can mark this question. We will call you to make an appointment to discuss and explain the unclear questions. Your comments will be treated confidentially according to the the Medical Research Involving Human Subjects Act (WMO), subject 12. If you have any questions, please contact Chantal Huisman (copdonderzoek@hu.nl or 06-14328209).

Name: …………………………………………………………………………………...

Date of filling out the questionnaire: ……………………………………………

Number of months you used the smartphone with application: ………….

……………………………………………………………………………………………..

Did you possess a smartphone before the start of the research? Yes / No

Did you purchase a smartphone after the research period? Yes / No

In the first part of the questionnaire you can answer the questions by using a scale from 0 to 7. See below the first statement:

“The smartphone was easy to use”
Totally disagree   0   1   2   3   4   5   6   7   Completely agree

If it was easy for you to use the smartphone you can circle the 7, and when it was very difficult for you to use the smartphone you circle the 0. If it is somewhere in between you can circle 1-6. The higher the number, the more you agree with the statement.
Please answer this question now.

<u>Instructions:</u> think back to the days when you used the smartphone with de application. Read the following statements carefully. Circle the number which matches your experience most.

Usefulness

1) The application supports me in an effective way.

   Totally disagree   0   1   2   3   4   5   6   7   Completely agree

2) The application helps me to have a healthy physical activity pattern.

   Totally disagree   0   1   2   3   4   5   6   7   Completely agree

Remark(s) for question 2:…………………………………………………………
…………………………………………………………………………………………
…………………………………………………………………………………………
…………………………………………………………………………………………
…………………………………………………………………………………

3) The application is useful.

Totally disagree 0 1 2 3 4 5 6 7 Completely agree

4) The application gives me control over the physical activities in my life.

Totally disagree 0 1 2 3 4 5 6 7 Completely agree

5) I can do the things that I want more easily.

Totally disagree 0 1 2 3 4 5 6 7 Completely agree

6) I save time by using the application.

Totally disagree 0 1 2 3 4 5 6 7 Completely agree

7) The application meets my needs.

Totally disagree 0 1 2 3 4 5 6 7 Completely agree

8) The application works as it should.

Totally disagree 0 1 2 3 4 5 6 7 Completely agree

Ease of use

9) The application is easy to use.

Totally disagree 0 1 2 3 4 5 6 7 Completely agree

10) I don't have to tap the screen of the smartphone often to achieve what I want.

Totally disagree 0 1 2 3 4 5 6 7 Completely agree

11) The application is flexible.

Totally disagree 0 1 2 3 4 5 6 7 Completely agree

12) It takes effort to use the application.

Totally disagree 0 1 2 3 4 5 6 7 Completely agree

Remark(s) for question 12: …………………………………………………………
………………………………………………………………………………………………
………………………………………………………………………………………………
………………………………………………………………………………………………
……………………………………………………………………………………

13) I can use the application **<u>without</u>** the written instructions.

Totally disagree 0 1 2 3 4 5 6 7 Completely agree

14) The application is easy to use.

Totally disagree 0 1 2 3 4 5 6 7 Completely agree

15) It is necessary to receive a training on the use of the application.

Totally disagree 0 1 2 3 4 5 6 7 Completely agree

16) The **smartphone** experienced technical failures.

Totally disagree 0 1 2 3 4 5 6 7 Completely agree

17) The **application** experienced technical failures.

Totally disagree 0 1 2 3 4 5 6 7 Completely agree

18) There are things that contradicted each other when I used the application.

Totally disagree 0 1 2 3 4 5 6 7 Completely agree

19) The application is user friendly.

Totally disagree 0 1 2 3 4 5 6 7 Completely agree

20) The application is easy to use.

Totally disagree 0 1 2 3 4 5 6 7 Completely agree

21) Both people that use the application occasionally, and people that use the application regularly will find it pleasant.

Totally disagree 0 1 2 3 4 5 6 7 Completely agree

Remark(s) for question 21: ……………………………………………………
………………………………………………………………………………………
………………………………………………………………………………………
………………………………………………………………………………………
……………………………………………………………………………

22) I can restore mistakes easily.

Totally disagree 0 1 2 3 4 5 6 7 Completely agree

23) I can use the application successful.

Totally disagree 0 1 2 3 4 5 6 7 Completely agree

24) I **didn’t** reach my goal because I forgot the smartphone.

Totally disagree 0 1 2 3 4 5 6 7 Completely agree

Remark(s) for question 24:………………………………………………………
………………………………………………………………………………………
………………………………………………………………………………………
………………………………………………………………………………………
……………………………………………………………………………

25) The battery of the smartphone was drained fast.

Totally disagree 0 1 2 3 4 5 6 7 Completely agree

Remarks for question 25: …………………………………………………………
……………………………………………………………………………………………
……………………………………………………………………………………………
……………………………………………………………………………………………
………………………………………………………………………………….

Ease of learning

26) I have learned to work with the application quickly.

Totally disagree 0 1 2 3 4 5 6 7 Completely agree

27) I can easily remember how to use the application.

Totally disagree 0 1 2 3 4 5 6 7 Completely agree

28) It is easy to learn how to work with the application.

Totally disagree 0 1 2 3 4 5 6 7 Completely agree

29) I was quickly apt to work with the application.

Totally disagree 0 1 2 3 4 5 6 7 Completely agree

Satisfaction

30) I am satisfied with the application.

Totally disagree 0 1 2 3 4 5 6 7 Completely agree

31) I will recommend the application to a friend.

Totally disagree 0 1 2 3 4 5 6 7 Completely agree

32) It is fun working with the application.

Totally disagree 0 1 2 3 4 5 6 7 Completely agree

33) The application works how I think it should work.

Totally disagree 0 1 2 3 4 5 6 7 Completely agree

34) The application is great.

Totally disagree 0 1 2 3 4 5 6 7 Completely agree

35) The application is in line with my needs.

Totally disagree 0 1 2 3 4 5 6 7 Completely agree

36) Use of the application is pleasant.

Totally disagree 0 1 2 3 4 5 6 7 Completely agree

37) The application helps me to increase my physical activity.

Totally disagree 0 1 2 3 4 5 6 7 Completely agree

Remark(s) on question 37: ………………………………………………………<br>……………………………………………………………………………………………<br>……………………………………………………………………………………………<br>……………………………………………………………………………………………<br>………………………………………………………………………………

38) By using the application I felt more powerful.

Totally disagree 0 1 2 3 4 5 6 7 Completely agree

In the second part of the questionnaire you will have to answer on a scale of 1-7 (instead of 0-7). The higher the number, the more you agree with the statement.

Privacy

39) It is comforting to know that the application continuously collects information about my physical activities.

Totally disagree 1 2 3 4 5 6 7 Completely agree

40) I **<u>don't</u>** think it is a problem that the physiotherapist can see information about my physical activities.

Totally disagree 1 2 3 4 5 6 7 Completely agree

41) I **<u>don't</u>** think it is a problem that my family can see information about my physical activities.

Totally disagree 1 2 3 4 5 6 7 Completely agree

42) I'm worried that the information could end up in the wrong hands.

Totally disagree 1 2 3 4 5 6 7 Completely agree

43) I **<u>don't</u>** think it is a problem that a good friend can see the information.

Totally disagree 1 2 3 4 5 6 7 Completely agree

44) I **<u>don't</u>** think it is a problem that selected family members can see the information.

Totally disagree 1 2 3 4 5 6 7 Completely agree

45) I **<u>don't</u>** think it is a problem that the health care insurance company can see the information.

Totally disagree 1 2 3 4 5 6 7 Completely agree

46) I **<u>don't</u>** think it is a problem that the government can see the information.

Totally disagree 1 2 3 4 5 6 7 Completely agree

47) I **<u>don’t</u>** think it is a problem that the information is stored forever.

Totally disagree 1 2 3 4 5 6 7 Completely agree

48) I **<u>don’t</u>** think it is a problem that the information is stored in medical files.

Totally disagree 1 2 3 4 5 6 7 Completely agree

49) Messages on the application should be shown to me only.

Totally disagree 1 2 3 4 5 6 7 Completely agree

50) I worry that I will lose the smartphone and others will get access to my information.

Totally disagree 1 2 3 4 5 6 7 Completely agree

51) I want to decide who gets to receive the information.

Totally disagree 1 2 3 4 5 6 7 Completely agree

52) I think the application is an infringement of my privacy.

Totally disagree 1 2 3 4 5 6 7 Completely agree

Feedback

53) I liked that I could see the information about my physical activities on the screen of the smartphone.

Totally disagree 1 2 3 4 5 6 7 Completely agree

54) Information about my physical activities does **not** need to be shown to me, this information is **only** for the caregiver.

Totally disagree 1 2 3 4 5 6 7 Completely agree

The figures below support the following questions.

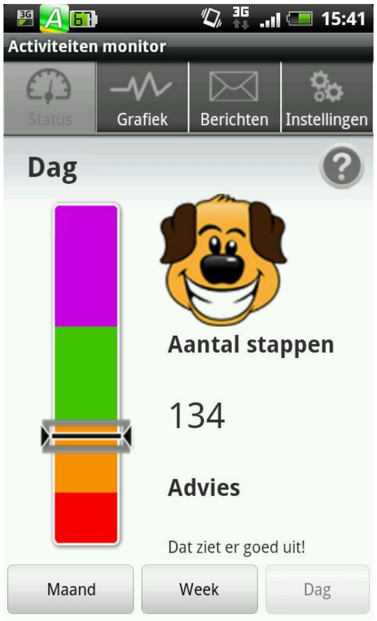

Figure 1:
Tab status in the application. The bar left, the emoticon right, number of steps and the advice. **[question 55-59]**

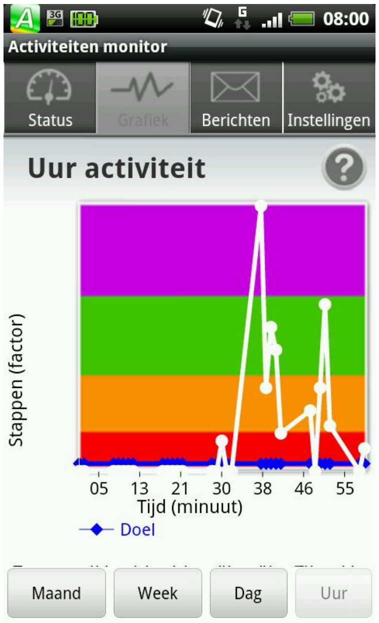

Figure 2:
Tab graph **[question 60]**

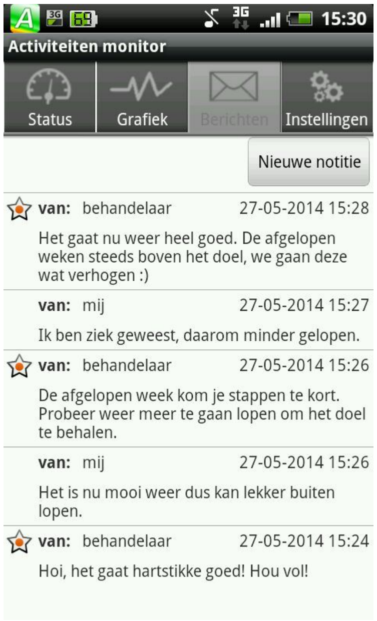

Figure 3:
Tab messages, text messages from en to the physiotherapist **[question 62]**

55) It was nice my physical activities were shown as number of steps [figure 1].

Totally disagree 1 2 3 4 5 6 7 Completely agree

56) The bar was a clear representation of how far I was towards reaching my step goal [see figure 1].

Totally disagree 1 2 3 4 5 6 7 Completely agree

Remark(s) for question 56: ……………………………………………………… ………………………………………………………………………………………… ………………………………………………………………………………………… ………………………………………………………………………………………… ………………………………………………………………………………

57) The emoticon, for example the dog, was stimulating [see figure 1].

Totally disagree 1 2 3 4 5 6 7 Completely agree

Remark(s) for question 57: ……………………………………………………… ………………………………………………………………………………………… ………………………………………………………………………………………… ………………………………………………………………………………………… ………………………………………………………………………………

58) It was pleasurable to have the option to choose different emoticons.

Totally disagree 1 2 3 4 5 6 7 Completely agree

59) The written advice was stimulating [see figure 1].

Totally disagree 1 2 3 4 5 6 7 Completely agree

Remark(s) for question 59: …………………………………………………………
……………………………………………………………………………………………
……………………………………………………………………………………………
……………………………………………………………………………………………
……………………………………………………………………………………

60) The graph gave me additional insight into my physical activities [see figure 2].

Totally disagree 1 2 3 4 5 6 7 Completely agree

Remark(s) for question 60: …………………………………………………………
……………………………………………………………………………………………
……………………………………………………………………………………………
……………………………………………………………………………………………
……………………………………………………………………………………

61) It was nice to see I reached my activity goal.

Totally disagree 1 2 3 4 5 6 7 Completely agree

Remark(s) for question 61: ……………………………………………………… …………………………………………………………………………………………… …………………………………………………………………………………………… …………………………………………………………………………………………… ……………………………………………………………………………………

62) I received messages **<u>from</u>** the physiotherapist on the application [see figure 3].

Never 1 2 3 4 5 6 7 Very often

63) I have sent messages **<u>to</u>** the physiotherapist by using the application [see figure 3].

Never 1 2 3 4 5 6 7 Very often

64) The messages from the physiotherapist support to reach my activity goal [see figure 3].

Totally disagree 1 2 3 4 5 6 7 Completely agree

Remark(s) on question 64: ……………………………………………………… …………………………………………………………………………………………… …………………………………………………………………………………………… …………………………………………………………………………………………… ……………………………………………………………………………………

65) The pictures on the home screen of the smartphone (emoticon, like dog and the traffic light colors) clearly stated the current status of my physical activities [see figure below].

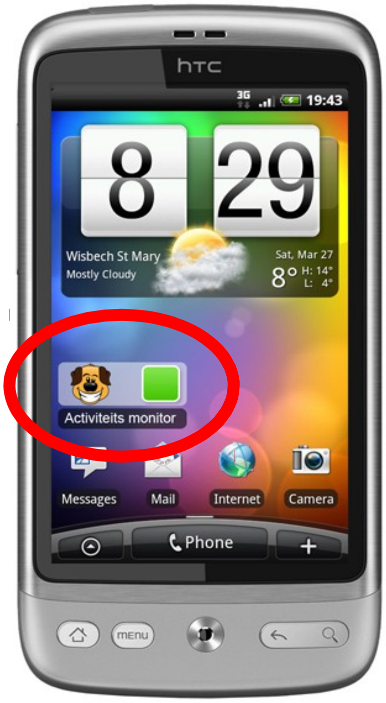

Totally disagree 1 2 3 4 5 6 7 Completely agree

Remark(s) on question 65: …………………………………………………………   ………………………………………………………………………………………………   ………………………………………………………………………………………………   ………………………………………………………………………………………………   ………………………………………………………………………………………

66) These aspects of the application were unnecessary, could be better, were unclear:

Remark(s) on question 66: …....…………………………………………………… ……………………………………………………………………………………………… ……………………………………………………………………………………………… ……………………………………………………………………………………………… ……………………………………………………………………………………………… ……………………………………………………………………………………………… ………………………………………………………………………………

67) These aspects in the application were nice, useful, clear:

Remark(s) on question 67: ……………………………………………………… ………………….. ……………………………………………………………………………………………… ……………………………………………………………………………………………… ……………………………………………………………………………………………… ……………………………………………………………………………………………… ……………………………………………………………

In the last part of the questionnaire you will have answer from 1 to 7 with '**never**' (1) on the left side and '**always**' (7) on the right side.

Circumstances

68) As a result of a bad health status I **couldn't** reach my activity goal.

Never 1 2 3 4 5 6 7 Always

Remark(s) on question 68: …………………………………………………
………………………………………………………………………………………
………………………………………………………………………………………
………………………………………………………………………………………
……………………………………………………………………………

69) I felt too tired to reach my activity goal.

Never 1 2 3 4 5 6 7 Always

Remark(s) on question 69: …………………………………………………
………………………………………………………………………………………
………………………………………………………………………………………
………………………………………………………………………………………
……………………………………………………………………………

70) I **couldn't** reach my activity goal because of personal circumstances.

Never 1 2 3 4 5 6 7 Always

71) I was too busy to reach my activity goal.

Never 1 2 3 4 5 6 7 Always

72) I quit smoking during the research period………. Yes / No……….

If the answer is yes, when did you quit? (date) ……………………..………………...
……………………………………………………………………………………………. If the answer is no, how many cigarettes do you smoke per day?
………………………………………………………………………………….
No, I smoked no longer/ I have never smoked (tick if applicable)…………......
……………………………………………………………………………………………

73) I would like to use the application again so I can start using it again …Yes / No …..

Thank you very much for completing the questionnaire!

Remarks:

…………………………………………………………………………………………………

…………………………………………………………………………………………………

…………………………………………………………………………………………………

…………………………………………………………………………………………………

…………………………………………………………………………………………………

…………………………………………………………………………………………………

…………………………………………………………………………………………………

…………………………………………………………………………………………………

…………………………………………………………………………………………………

………………………………………………………………...

You will soon be called by Chantal Huisman.
