# Supplementary material for: Perceptions of Patients With Chronic Obstructive Pulmonary Disease and Their Physiotherapists Regarding the Use of an eHealth Intervention
Source: JMIR Hum Factors. 2017 Sep 19;4(3):e20. doi: 10.2196/humanfactors.7196 (PMC5627045; doi:10.2196/humanfactors.7196)
Supplement: Multimedia Appendix 4 [file humanfactors_v4i3e20_app4.pdf]

Multimedia Appendix 4

Results patient questionnaire

Table 1. Results on use. Scores range from 0-7 with 0: totally disagree and 7: totally agree. Statements are arranged on degree of agreeability.

question	Statement	Mean ± standard deviation
20	The application is easy to use	5.95 ± 1.32
37	The application helped me to increase my PA	4.92 ± 2.2
38	The application made me feel more fit	4.32 ± 2.18
15	A training on the use of the application is necessary	3.25 ± 2.23
25	Battery life of the phone was short	3.15 ± 2.67
16	The smartphone suffered from technical failure	1.77 ± 2.37
17	The application suffered from technical failure	1.37 ± 1.93
24	I did not reach my PA goal because I forgot my phone	1 ± 1.43

Table 2. Results on privacy. Scores range from 1-7 with 1: totally disagree and 7: totally agree. Statements are arranged on degree of agreeability.

Question	Statement	Mean ± standard deviation
	I do **not** mind that the following persons have access to my PA information:	
40	• PT	6.48 ± 0.85
41	• Family	5.77 ± 1.72
44	• Family members I selected	5.72 ± 1.88
45	• Care insurers	4.33 ± 2.57
43	• A good friend	4.23 ± 2.34
46	• Local authority	3.25 ± 2.38
39	It is good to know the application continuously collects data on my PA	5.92 ± 1.34
51	I want to determine which PA information is sent to whom	5.52 ± 2.03
48	I do **not** mind that my PA information is saved in a medical file	5.47 ± 2
47	I do **not** mind that my PA information is saved forever	4.48 ± 2.53
50	I worry that I lose my phone and others can access my PA information	4.03 ± 2.22
49	Messages in the application should only be visible for me	4 ± 2.12
52	The application is an invasion on my privacy	2.65 ± 2.06
42	I worry that this information ends up in the wrong hands	2.73 ± 2.03

Table 3. Results on feedback preferences. Scores range from 1-7 with 1: totally disagree and 7: totally agree. For question 62 and 63 scores were 1: never to 7: very frequently. Statements are arranged on degree of agreeability.

Question	Statement	Mean ± standard deviation
61	It was rewarding to see that I reached my PA goal	6.3 ± 1.31
55	I liked the fact that PA was presented in steps	6.28 ± 1.39
53	I liked the fact that I could see my PA information on the smartphone	6.23 ± 1.14
56	The bar clearly showed my PA goal progress	6.22 ± 1.39
60	The graph provided extra insight into my PA	5.63 ± 1.74
65	The widget on the home screen of the smartphone clearly stated my PA status	5.6 ± 1.84
57	The emoticon was stimulating	5.25 ± 2.16
59	The written advice was stimulating	5.15 ± 1.81
58	It was pleasant to have various choices of emoticons	4.87 ± 1.89
64	The messages from the PT supported me in reaching my PA goal	3.34 ± 2.35
62	I received messages from the PT in the application	3.1 ± 2.15
63	I sent messages to the PT in the application	2.05 ± 1.71
54	My PA information should not be visible to me, only to my health care professional	1.68 ± 1.49

Table 4. Results on personal circumstances. Scores range from 1-7 with 1: never and 7: always. Statements are arranged on frequency of occurrence.

question	Statement	Mean ± standard deviation
68	I did not reach my PA goal because of my state of health	2.39 ± 1.33
69	I was too tired to be able to reach my PA goal	2.28 ± 1.49
70	I did not reach my PA goal because of personal circumstances	1.73 ± 1.21
71	I was too busy to reach my PA goal	1.68 ± 1.26
